# Let referees see the data

**DOI:** 10.1038/sdata.2016.33

**Published:** 2016-05-10

**Keywords:** Peer review, Research data

As the scientific community pushes for data to be better incorporated into and credited within the scholarly literature, concerns have reasonably been raised that review of data could further stress an over-burdened peer-review system.

Our experience so far at *Scientific Data*, however, has demonstrated that data *can be* evaluated efficiently during peer review, in a timely and constructive manner, and that exposing data to reviewers need not lead to unreasonable burdens.

*Scientific Data’s* publications differ from traditional research articles in that they serve to help others use, access and understand data, rather than presenting specific claims. Assessment therefore cannot rest on traditional notions of ‘conclusiveness’ or ‘significance’. Instead, our peer-review process seeks to determine whether the data being described are sound and include sufficient context to enable wider use by the community.

Our instructions to referees, in essence, ask them to determine whether they would use a dataset in the kinds of applications outlined by the authors. We find that for appropriate subject experts this quickly implies a very concrete set of criteria—scientists who work with complex data know what they need to see before they will trust a dataset. Referees are further guided by a set of eight questions that help focus evaluation on data quality and reusability (http://go.nature.com/jvzyKV). For a more in-depth exploration of the conceptual aims of data peer review we refer readers to Lawrence *et al.*^1^

We believe that our referees must be able to view the actual data, so we will not send a manuscript for evaluation until the underlying data can be accessed easily and securely. Links to the data records are tested and added to a cover page attached to the manuscript PDF. This process helps us filter out incomplete or preliminary submissions at an early stage, and ensures that referees have the information they need to supply a detailed report as soon they receive the manuscript—no time is wasted requesting access to data. We feel that this promotes an objective and constructive evaluation, and in some cases even helps streamline review. Indeed, more than ninety percent of the manuscripts sent out for peer review at *Scientific Data* are ultimately accepted for publication, and the majority goes through only one round of revision.

Authors are asked to provide their data in the ‘rawest’ form that will support reuse, without substantial filtering or processing that imprints a particular interpretation on the data. We encourage authors to consider releasing data at multiple levels when this would enable broader reuse—for example, proteomics data may best be released as ‘raw’ spectra as well as more processed peptide- or protein-level measurements. Referees play an important role in assessing whether data have been provided in an appropriate manner, and help us interpret these policies in line with community standards.

It is common for our referees to comment on the actual data in their reports, and their feedback often leads to material improvements in things like data formatting or presentation. Some referees will even analyse or create visualizations from the data, while preparing their reports.

During this process, referees do occasionally identify serious issues that would not have been apparent from an assessment of the manuscript alone. Typical pitfalls include incomplete deposition of the underlying data, or evidence of poor data quality. Often these issues can be addressed by authors, for example by cleaning errors in the data or by acknowledging caveats in the dataset more clearly in the manuscript. But sometimes evaluation reveals fundamental issues that cannot easily be addressed, and we must decline publication.

From a sample of eleven papers submitted to the journal and rejected after peer review, we observed that seven decisions (64%) were based, in whole or in part, on issues that emerged from referees’ comments on the actual data. Rejections remain relatively rare at the journal, but we feel these numbers provide compelling, if anecdotal, support for the idea that exposing data to referees can improve the assessment process.

We do not, however, expect or require our referees to assess all of the data in detail. Detailed curation of data is best performed by community data repositories with expert staff, which is why we collaborate with and support more than eighty different data repositories (http://go.nature.com/jPM768). Our in-house data curation editor manages these relationships, and provides an extra pair of expert eyes to ensure the integrity of data published at the journal.

This multi-level assessment approach—combining traditional peer review with in-house curation and repository expertise—also helps us ensure that the data we publish are as ‘FAIR’ as possible^2^. The FAIR principles for effective data sharing encourage researchers to make their data Findable, Accessible, Interoperable, and Reusable. Technical quality, assessed through our peer-review process, is essential for reusability. Findability and interoperability are, though, equally important, and can often be better implemented by specialist repository staff and curators.

In parallel, we are working to improve how we evaluate types of data that pose inherent challenges for peer review. Dynamic datasets are one such example. Here, we ask that a snapshot of the data be permanently archived and referenced in the manuscript—helping record exactly what referees assessed, even as the dataset grows or evolves. Clinical research datasets also present challenges for anonymous peer review because special restrictions on data access are often needed to protect participant privacy. To help overcome these barriers, we are developing a series of guidelines designed to make wider publication and peer review of clinical data practical^3^.

Data-focused journals, like *Scientific Data*, bear a special responsibility to peer review data effectively. But, overall, our experience suggests that making data more easily available to referees could aid assessment at other journals, and help combat irreproducibility in the scholarly literature.
